# The Involvement of Service Users and People With Lived Experience in Mental Health Care Innovation Through Design: Systematic Review

**DOI:** 10.2196/46590

**Published:** 2023-07-25

**Authors:** Lars Veldmeijer, Gijs Terlouw, Jim Van Os, Olga Van Dijk, Job Van 't Veer, Nynke Boonstra

**Affiliations:** 1 Department of Psychiatry Utrecht University Medical Center Utrecht Netherlands; 2 Department of Healthcare and Welfare NHL Stenden University of Applied Sciences Leeuwarden Netherlands; 3 NHL Stenden Library NHL Stenden University of Applied Sciences Leeuwarden Netherlands; 4 KieN VIP Mental Health Care Services Leeuwarden Netherlands

**Keywords:** design approaches, innovation, psychiatry, mental health care, involvement, service users, people with lived experience, cocreation, mobile phone

## Abstract

**Background:**

Mental health care faces challenges that not only necessitate innovation but also require the involvement of service users and people with lived experience in developing and evaluating mental health care services. As the development of digital interventions is becoming more prevalent, design approaches are increasingly finding their way into mental health. There is evidence that these approaches can successfully integrate user experience into mental health services. However, there is no clear overview of the studies conducted and the lessons learned concerning the involvement of service users and people with lived experience.

**Objective:**

In this systematic review, we aimed to provide an overview of the involvement of service users and people with lived experience in mental health care services through design approaches and to synthesize the advantages of design approaches in mental health care.

**Methods:**

The following 5 databases were searched for relevant abstracts: PsycINFO, PubMed, Web of Science, Scopus, and Embase. In addition, 2 health design journal archives, *Design for Health* and *The Journal of Health Design*, were searched. To categorize the results, we collected the reported added value from the included articles and conducted a thematic synthesis in which the themes were developed from the retrieved data. The themes were discussed, revised, and checked until saturation was achieved.

**Results:**

We included and categorized 33 papers. Most studies involved service users, primarily adults, and used various design approaches. Most of these studies aimed to design or evaluate digital interventions. Service users and people with lived experience were involved in different roles but never as decision makers. Studies that used co-design approaches exhibited the highest levels of involvement. Various added values were reported, including tailoring and testing interventions and digital interventions, improving engagement and collaboration, gathering the needs of stakeholders, and empowering participants as resourceful actors. The challenges reported were maintaining participants’ continued participation throughout the study, managing the iterative nature of design, providing a safe space, balancing insights from design and medical science, and navigating design processes in medical environments.

**Conclusions:**

This systematic review provides an overview of the studies that used design approaches to involve service users and people with lived experience in mental health care innovation. Design approaches have advantages in mental health care innovation, offering added value and having manageable challenges. Future studies using design approaches in mental health care should involve participants as partners and decision makers and report on collaboration in a systematic and clear manner.

## Introduction

### Background

Mental health care services are in need of transformation [1]. However, incremental and iterative integration of advances is recommended rather than a complete paradigm shift [2]. Creative thinking [3] and the involvement of people with lived experience [4] are needed to catalyze these advances. Design and design research hold the potential to drive these incremental and iterative changes in traditional mental health care services, as it builds on creative thinking and doing [5]. Over the last decade, the application of design approaches in mental health care services has increased and has shown to accelerate innovation [6-8]. For example, design or co-design has been effective in designing or redesigning psychiatric facilities [9-11] and has proven successful in engaging end users in the design process of novel therapies [12-14]. The integration of design into mental health services is a logical continuation of the existing solutions to the challenges of the sector. These challenges include the relationship between professionals and patients in the era of value-based competition in health care [15,16], paradigm shifts in the diagnosis of mental health conditions [2,17,18], the integration of empirical evidence of recovery into traditional services [19,20], the efficacy of psychotherapies and pharmacotherapies in adults [21], and the cocreation of novel concepts and language with people with lived experience [22].

### Design Approaches

Design can contribute to change and innovation in mental health care because it is a transformative discipline [23]. A scoping review of the extensive evidence on the role of the arts in improving health and well-being concluded that design, as a subcategory of the arts, can help prevent the onset of mental health conditions and support their treatment [24]. Design differs from the rational way of solving problems, that is, designers do not aim for the optimal solution to a given problem but choose a more exploratory approach, where the problem and solution evolve together [25]. Although digital innovation in mental health care has shown promising results, such as ecological momentary assessment and passive sensor tracking [26], the field encounters challenges involving service users and people with lived experience in the design of digital health interventions [27]. Despite these challenges, the involvement of service users and people with lived experience in design is seen as essential to tailor innovations to their needs and balance the power between patients and professionals [4]. Recent developments in design methodologies offer potential approaches to address these issues. For example, in recent years, the scope of design in health has been expanded by introducing various user-centered approaches, such as co-design [28], experience-based co-design [29], participatory design [14], design thinking [8], design research [30], iterative design [13], value-sensitive design [31], experience design [32], and human-centered design [33]. In these design approaches, designers aim to integrate users’ tacit knowledge into the design of products and services by assigning them an important role in the design process [34]. These design approaches use various methods from other fields [35], such as natural sciences, sociology, psychology, anthropology, and visual arts [36]. Design approaches have provided opportunities to all the stakeholders involved in the innovation processes to discover the user experience and to place the end users at the center of the design process by involving them as stakeholders [37].

### Level of Involvement of Service Users and People With Lived Experience

There is a continuing focus on how service users and people with lived experience can be involved in mental health projects. A recently developed framework, The Involvement Matrix [38], was cocreated with experts by experience and researchers. The Involvement Matrix describes 5 roles (ie, listener, cothinker, adviser, partner, and decision maker) and 3 phases. Mainly, these roles are relevant to assessing the level of involvement of people with lived experience and service users in studies using a design approach. The lowest level of involvement is “the listener,” as they only receive information, whereas the second level, “the cothinker,” is also asked for an opinion. The third level, “the adviser,” gives solicited or unsolicited advice, whereas on the fourth level, “the partner,” acts as an equal partner. The “decision maker” can be seen as having the highest level of involvement, as the decision maker takes the initiative and is involved in the (final) decision. Although design approaches are useful in engaging stakeholders in mental health care innovation projects, there is currently no comprehensive overview of the studies that used design approaches, including which specific approaches were used, the roles service users and individuals with lived experience had, and the added value they generated.

### Objective

In this systematic review, we aimed to provide an overview of the involvement of service users and people with lived experience in mental health care services through design approaches and to synthesize the advantages of design approaches in mental health care.

## Methods

### Databases and Search Strategy

The following 5 databases were searched for relevant abstracts: PsycINFO, PubMed, Web of Science, Scopus, and Embase. These databases cover a wide range of published studies in the field of health and design. *Design for Health* and *The Journal of Health Design*, which are both health design journals, were also searched because they publish papers at the intersection of health and design. The terms used for the PubMed search are presented in Textbox 1.

Owing to the differences in search engine functionality, the method by which the terms were entered differed per database. A complete overview of these terms is provided in Multimedia Appendix 1. Before conducting the definitive search, we contacted an information specialist and performed 4 preliminary searches by using different terms to minimize the possibility of missing relevant studies. We followed the PRISMA (Preferred Reporting Items for Systematic Reviews and Meta-Analyses) guidelines [39] to report this review. The search was conducted between September 6, 2022, and October 28, 2022.

**Terms used for searching PubMed.**

A combination of search terms were used to identify relevant papers under the following categories: ((“Mental Health”[Mesh] OR mental-health[tiab] OR mental-hygiene[tiab] OR mental-care[tiab] OR “Psychiatry”[Mesh] OR psychiatr*[tiab]) AND (user-centered development*[tiab] OR user-centred design*[tiab] OR user-centred development*[tiab] OR user-centric design*[tiab] OR user-driven design*[tiab] OR user-driven development*[tiab] OR “User-Centered Design”[Mesh] OR user-centered-design*[tiab] OR usability-testing[tiab] OR Co-design*[tiab] OR Participatory-design*[tiab] OR Experience-based-co-design*[tiab] OR Interaction-design*[tiab] OR Service-design*[tiab] OR Systemic-design*[tiab] OR Patient-centered-design*[tiab] OR Human-centered-design*[tiab] OR Value-sensitive-design*[tiab] OR Design-thinking[tiab] OR Design-research*[tiab] OR Design-method*[tiab] OR Design-session*[tiab] OR design-approach*[tiab] OR design-principle*[tiab] OR design-choice*[tiab] OR universal-design*[tiab] OR Creativ-method*[tiab] OR Creative-session*[tiab] OR Generative-design*[tiab] OR Generative-method*[tiab] OR Generative-session*[tiab] OR Iterative-design*[tiab] OR Design-driven-innovation*[tiab] OR Speculative-design*[tiab] OR Critical-design*[tiab] OR Discursive-design*[tiab] OR Product-design*[tiab])) NOT (“Research Design”[Mesh] OR research-design[tiab] OR research-designs[tiab] OR research-protocol*[tiab] OR research-instrument*[tiab] OR study-design[tiab] OR study-designs[tiab] OR research-method*[tiab] OR methodology[tiab] OR methodological-research[tiab])

### Study Selection and Inclusion and Exclusion Criteria

We included studies that discussed the involvement of service users and people with lived experience in mental health care services through design approaches. We included only original reports or papers that (1) involved service users, people with lived experience, or both; (2) mentioned design approaches; (3) involved an empirical study; and (4) conducted the study in settings including mental health care service or psychiatry programs. Papers that met these criteria were selected for full-text screening. We defined service users as participants who used mental health care services at the time of their involvement and people with lived experience as those who had used mental health care services in the past but were not currently using them during the study in which they were involved. We included empirical studies because they could provide insights into the level of involvement. We selected studies conducted in mental health care and psychiatry care service settings to retrieve as many studies as possible. We are aware that there is an overlap between the terms and that the services offered under both contexts may vary across countries.

The following exclusion criteria were used for full-text screening: (1) non–peer-reviewed papers such as abstracts, conference posters, or trade journals; (2) papers with full texts not available; (3) papers in languages other than English; (4) monographs or short reports; and (5) papers with insufficient information in the abstract.

### Research Questions

To provide an overview of the involvement of service users and people with lived experience in mental health care services through design approaches and to provide insight into the advantages of design approaches in mental health care innovation, the included studies were analyzed using the following research questions (RQs):

- RQ 1: Who were included (service users, people with lived experience, or both)?
- RQ 2: What design approach was used?
- RQ 3: What was the aim of the innovation?
- RQ 4: What were the roles of service users and people with lived experience?
- RQ 5: What was the added value of involving service users and people with lived experience through design approaches?
- RQ 6: What were the challenges in involving service users and people with lived experience through design approaches?

### Screening Process and Study Selection

After removing duplicates, the titles and abstracts of the papers were screened using Rayyan (**Rayyan** Systems Inc) [40]. A total of 2 reviewers (LV and GT) independently reviewed all the titles and abstracts and were double blinded for relevance with the formulated inclusion and exclusion criteria. Papers were only included if both LV and GT agreed, and a plausible argumentation for consideration of inclusion always led to the inclusion. Full-text papers were retrieved after this step. During the full-text screening phase, the first 20% of the papers were randomly selected and double-blind reviewed by 2 reviewers (LV and GT). The random selection ensured that the screening process was unbiased, and the double-blind review increased its reliability. Subsequently, the primary reviewer (LV) reviewed the other included papers for full-text reading, which helped to maintain consistency throughout the review process.

### Data Extraction

Data were extracted using structured forms, including the characteristics of participants (service users and people with lived experience), aim of innovation, design approach, and role of the participants in the design approach. In addition, we focused on the added value and challenges of involving service users and people with lived experience through a design approach that the authors reported in the included studies. Findings from all the studies regarding the reported added values and challenges were extracted and collated using a thematic synthesis. Thematic synthesis preserves principles that have traditionally been important to systematic reviewing [41]. This synthesis was performed by LV and GT. Because our primary aim was to find all possible added values reported (ie, a comprehensive overview), we refrained from using the existing design frameworks to guide data extraction and analysis, as such an approach may have impeded the identification of novel findings that were not aligned with the themes these frameworks provide or other a priori themes that we could have developed [42]. As such, the themes that emerged from the data were discussed and revised to minimize overlap and were checked by researchers JvV and NB. This process was repeated until saturation of the themes was achieved.

## Results

### Search Results

**Figure 1:**
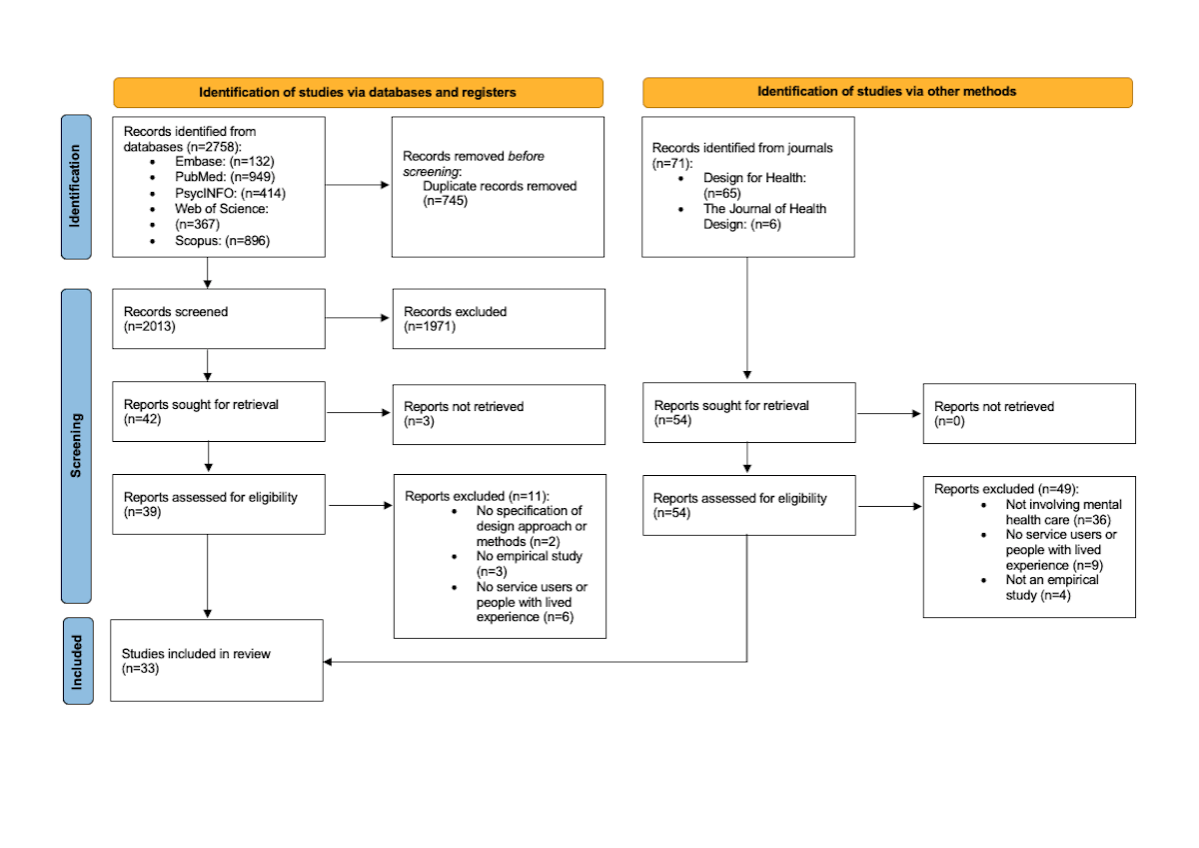
Selection process: PRISMA (Preferred Reporting Items for Systematic Reviews and Meta-Analyses) 2020 flow diagram.

Our initial database search yielded 2758 records. After removing 745 (27.01%) duplicates from 2758 records, the titles and abstracts of the 2013 (72.99%) records were screened. Next, excluding 1971 (97.91%) papers from the 2013 records after full-text screening, 42 (2.13%) records were sought for retrieval, and finally, 28 (67%) papers were included. We also identified 71 records through a journal search. After removing 17 (24%) duplicates from 71 records, we retrieved 54 (76%) records that were assessed for eligibility. Of these 54 records, 5 (9%) were included. This resulted in 33 included papers in this systematic review (Figure 1 shows a flow diagram of the results in the different selection stages). In both stages, a consensus was reached by the reviewers regarding the inclusion and analysis of the papers. All the included studies were published between 2010 and 2022. An overview of these studies is presented in Table 1. In Table 1, to avoid misinterpretation, we retained the terms that were used by the authors to describe their participants.

The following section outlines the characteristics of the included studies and the results orientated toward the RQs. First, we have described whether the studies included service users, people with lived experience, or both, as well as the age group of the involved participants. Then, we have summarized the aim of the innovation and reported the design approaches used. To address the level of involvement, we have presented the roles of service users and people with lived experiences. Finally, we have elaborated on the included studies to provide a context for the reported added value and challenges.

**Table 1:** Overview and categorization of the included studies.

Study, year	Study title	Participants and age group	Aim or aims of innovation	Approach and role
Owens et al [43], 2010	Involving service users in intervention design: a participatory approach to developing a text‐messaging intervention to reduce repetition of self‐harm	Service users Adults	Digital intervention development	Participatory workshops Cothinkers
Gammon et al [44], 2014	Service users’ perspectives in the design of an online tool for assisted self-help in mental health: a case study of implications	Service users Adults	Digital intervention development—recovery orientated	Community-based participatory research with iterative and cocreative design Cothinkers
Terp et al [45], 2016	A room for design: through participatory design young adults with schizophrenia become strong collaborators	Service users Adolescents	Digital intervention development	Co-design Partners
Grim et al [46], 2017	Development and usability testing of a web-based decision support for users and health professionals in psychiatric services	Service users People with lived experience Adults	Digital intervention development	Participatory design Cothinkers
Nakarada-Kordic et al [6], 2017	Co-designing for mental health: creative methods to engage young people experiencing psychosis	Service users Young people	Service improvement	Co-design Partners
Schmitt and Yarosh [47], 2018	Participatory design of technologies to support recovery from substance use disorders	Service users Adults	Digital intervention development—recovery orientated	Participatory design Cothinkers
McClelland and Fitzgerald [48], 2018	A participatory mobile application (app) development project with mental health service users and clinicians	Service users Not specified	Digital intervention development	Co-design Cothinkers
Vilardaga et al [49], 2018	User-centered design of learn to quit, a smoking cessation smartphone app for people with serious mental illness	Service users Adults	Digital intervention development	User-centered design Cothinkers
Terp et al [50], 2018	A smartphone app to foster power in the everyday management of living with schizophrenia: qualitative analysis of young adults’ perspectives	Service users Adults	Digital intervention development—recovery orientated	Participatory design Partners
Vieira da Silva and Bueno [51], 2018	Compass: a personal organization mobile app for individuals with mental disorders	Service users and people with lived experience Adults	Digital intervention development	User-centered design Cothinkers
Hackett et al [52], 2018	Co-designing for quality: creating a user-driven tool to improve quality in youth mental health services	Service users Young people	Digital intervention development	Experience-based co-design Advisers
Mulvale et al [53], 2019	Co-designing services for youth with mental health issues: novel elicitation approaches	Service users Young people	Service improvement	Co-design Advisers
Romm et al [54], 2019	Designing easy access to care for first-episode psychosis in complex organizations	Service users Adults	Service improvement	Service design Cothinkers
Derks et al [55], 2019	Development of an ambulatory biofeedback app to enhance emotional awareness in patients with borderline personality disorder: multicycle usability testing study	Service users Adults	Digital intervention development—recovery orientated	User-centered design Cothinkers
Realpe et al [56], 2019	Co-designing a virtual world with young people to deliver social cognition therapy in early psychosis	Service users and people with lived experience Young people	Digital intervention development—recovery orientated	Co-design Advisers
Maathuis et al [57], 2019	Exploring human values in the design of a web-based QoL^a^ instrument for people with mental health problems: a value-sensitive design approach	Service users Not specified	Digital intervention development	Value-sensitive design Cothinkers
Fonseka et al [58], 2019	Collaborating with individuals with lived experience to adapt CANMAT^b^ clinical depression guidelines into a patient treatment guide: the CHOICE-D^c^ co-design process	People with lived experience Adults	Service improvement—recovery orientated	Co-design Partners
Terlouw et al [59], 2020	Design of a digital comic creator (it’s me) to facilitate social skills training for children with autism spectrum disorder: design research approach	Service users Young people	Digital intervention development	Design research Advisers
Stawarz et al [60], 2020	Design considerations for the integrated delivery of cognitive behavioral therapy for depression: user-centered design study	Service users Adults	Digital intervention development	User-centered design Advisers
Callan et al [61], 2020	CBT^d^ MobileWork: user-centered development and testing of a mobile mental health application for depression	Service users Adults	Digital intervention development—recovery orientated	User-centered design Cothinkers
Flobak et al [62], 2021	Designing videos with and for adults with ADHD^e^ for an online intervention: participatory design study and thematic analysis of evaluation	People with lived experience Adults	Digital intervention development	Participatory design Cothinkers
van der Meer et al [63], 2021	Targeting personal recovery of people with complex mental health needs: the development of a psychosocial intervention through user-centered design	Service users Adults	Recovery orientated	User-centered design Advisers
García et al [64], 2021	Co-design of avatars to embody auditory hallucinations of patients with schizophrenia: a study on patients’ feeling of satisfaction and psychiatrists’ intention to adopt the technology	Service users Adults	Digital intervention development—recovery orientated	Co-design Advisers
Jonathan et al [65], 2021	A smartphone-based self-management intervention for bipolar disorder (livewell): user-centered development approach	Service users Adults	Digital intervention development—recovery orientated	User-centered development approach Cothinkers
Milton et al [66], 2021	Technology-enabled reform in a nontraditional mental health service for eating disorders: participatory design study	People with lived experience Not specified	Digital intervention development—recovery orientated	Participatory design Cothinkers
Sanin et al [67], 2021	Creative well-being. prototyping an arts-health practice program for mental health recovery	Service users Not specified	Service improvement—recovery orientated	Participatory design Autonomous design Partners
Knight et al [14], 2021	Participatory design to create a VR^f^ therapy for psychosis	People with lived experience Not specified	Digital intervention development	Participatory design Advisers
Kruzan et al [68], 2022	Centering lived experience in developing digital interventions for suicide and self-injurious behaviors: user-centered design approach	People with lived experience Adolescents	Digital intervention development—recovery orientated	User-centered design Advisers
Bos et al [69], 2022	A web-based application for personalized ecological momentary assessment in psychiatric care: user-centered development of the PETRA^g^ application	Service users Not specified	Digital intervention development	User-centered development approach Cothinkers
Bongers et al [70], 2022	I need to know: using the CeHRes^h^ roadmap to develop a treatment feedback tool for youngsters with mental health problems	People with lived experience Young people	Digital intervention development	Co-design Advisers
Wiberg et al [71], 2022	Internet-based cognitive behavior therapy for eating disorders—development and feasibility evaluation	Service users and people with lived experience Adults	Digital intervention development—recovery orientated	User-centered design Cothinkers
Illarregi et al [72], 2022	Is designing therapeutic? a case study exploring the experience of co-design and psychosis	Service user Adult	Intervention development	Co-design Partner
Jenness et al [73], 2022	Lessons learned from designing an asynchronous remote community approach for behavioral activation intervention for teens	Service users Teenagers and adolescents	Digital intervention development	Human-centered design Advisers

^a^QoL: quality of life.

^b^CANMAT: Canadian Network for Mood and Anxiety Treatments.

^c^CHOICE-D: Canadian Network for Mood and Anxiety Treatments Health Options for Integrated Care and Empowerment in Depression.

^d^CBT: cognitive behavioral therapy.

^e^ADHD: attention-deficit/hyperactivity disorder.

^f^VR: virtual reality.

^g^PETRA: Personalized Treatment by Real-time Assessment.

^h^CeHRes: Centre for eHealth Research roadmap.

### Characteristics of the Included Studies

All the included peer-reviewed articles were published between 2010 and 2022.

#### Participants

Of the 33 studies, 6 (18%) involved individuals with lived experience [14,58,62,66,68,70], 23 (70%) involved service users [6,43-45,47-50,52-55,57,59-61,63-65,67,69,72,73], and 4 (12%) involved both [46,51,56,71]. A total of 18 (55%) studies of the 33 studies included adult participants [43-47,49-51,54,55,58,60-65,71,72], 6 (18%) studies described their participants as young people [6,52,53,56,59,70], 3 (9%) studies described their participants as adolescents [45,68,73], and 6 (18%) did not specify the age group of their participants [14,48,57,66,67,69].

#### Aim of the Innovation

In total, 27 studies aimed to develop interventions, with 12 focusing on recovery [44,47,50,55,56,61,63-66,68,71], 3 targeting service improvement [6,53,54], and 2 targeting both [58,67]. A total of 26 studies focused on digital innovation design or evaluation [14,43-52,55-57,59-62,64-66,68-71,73] and 1 focused on design as an intervention itself [72].

#### Design Approaches

Co-design was used as the design approach in 9 studies [6,45,48,53,56,58,64,70,72]. User-centered design was used in 8 studies [49,51,55,60,61,63,68,71], and participatory design was used in 7 studies [14,46,47,50,62,66,67]. Other identified approaches included value-sensitive design [57], experience-based co-design [52], service design [54], design research [59], human-centered design [73], user-centered development [65,69], community-based participatory research [44], and participatory workshops [43].

#### Level of Involvement

The Involvement Matrix [38] was used to ascertain the roles of service users and individuals with lived experience in the analyzed studies. None of the studies included the participants as “listeners,” whereas 16 studies featured participants in the role of a “cothinker” [43,44,46-49,51,54,55,57,61,62,65,66,69,71], providing their opinions on ideas or evaluating test sessions of innovations developed by the researchers. In addition, participants served as “advisers” in 11 studies, offering solicited or unsolicited feedback to researchers and designers [14,52,53,56,59,60,63,64,68,70,73]. In 6 studies, participants functioned as equal “partners” with researchers and designers [6,45,50,58,67,72]. However, none of the studies identified participants in the decision maker role and reported involving participants in the final decision-making processes. The studies in which co-design was used as the approach exhibited the highest levels of participant involvement.

### Reported Added Values

#### Overview

The included studies reported a wide range of added value of the involvement of service users and people with lived experience through design approaches. Not every article mentioned specific design approach–related benefits [49,70]. On the basis of the thematic synthesis, we divided these added values into 4 categories.

#### Design for Tailoring and Testing (Digital) Applications and Interventions

In total, 6 studies reported on the added value of tailoring and testing existing designs with service users and people with lived experience [51,55,61,64,69,71]. Vieira da Silva and Bueno [51] adopted a user-centered design approach for the development and testing of the Compass app, which aimed to support people with mental health problems in their daily lives. The study found that patient testing provided key learnings for the final prototype, such as improvement of the interface design, resulting in the app meeting the users’ needs and expectations. Derks et al [55] cyclically tested an outpatient biofeedback application to increase emotional awareness in patients with borderline personality disorders. Testing with service users provided insights into the usability of the application. Wiberg et al [71] used a user-centered design process for the development and feasibility assessment of an internet-based cognitive behavioral therapy for patients with eating disorders. This approach contributed to improvements and adjustments to the program according to the end users’ needs and perceptions. Bos et al [69] described a user-centered development approach for a web-based application for personalized ecological momentary assessment in psychiatric care. The user-centered approach ensured that the developed application, which was tested with service users, was intuitive, user-friendly, and useful for clients and clinicians. García et al [64] co-designed avatars to embody auditory hallucinations. The co-design approach guaranteed that the service users found the developed software to be complete and useful for representing their voices. Callan et al [61] used an iterative design process to develop and test a mobile health app for individuals with depression, incorporating user feedback to improve the app’s appearance, navigation, content, and organization.

#### Design for Increasing Engagement and Collaboration in the Development of Interventions

We observed that 10 studies reported added value on increasing user engagement and collaboration in the development of interventions [14,52-54,58,59,65-68]. Romm et al [54] focused on the use of service design to address known barriers to developing early intervention services in complex health care organizations. By emphasizing cocreation and divergent thinking, service design encouraged experimentation and innovation to improve service development, engaging service users in organizational improvement. Jonathan et al [65] reported on a user-centered design process for developing a smartphone-based self-management intervention for individuals with bipolar disorder. This approach prioritized collaboration among stakeholders, which helped to personalize service users’ goals and plans and integrate human support as a self-management tool. Kruzan et al [68] presented a user-centered design approach to engage individuals with self-harming thoughts and behaviors who may be uncomfortable in traditional in-person therapy settings. User-centered design methods allow for flexibility in engaging individuals and facilitating collaboration in the most comfortable and accessible ways, such as through the internet. Sanin et al [67] collaborated with occupational therapists and inpatients to develop an arts-health program prototype. The cocreation of designers and occupational therapists generated a collaborative design process, expanding the creative practices of occupational therapists. Hackett et al [52] described the experience-based co-design process for improving the quality of youth mental health services. The co-design event emphasized communication and collaboration in delivering quality care, and the feedback tools developed by young participants served as an important starting point for continuous quality improvement.

In another article, Mulvale et al [53] highlighted the effectiveness of elicitation techniques such as experience maps, trigger videos, and prototyping in promoting mutual understanding and shared ideas for system changes. The experience-based co-design process, which prioritized the engagement of people with lived experience in the design of mental health services, was consistent with a recovery orientation that calls for collaboration with service users in the design of services. Terlouw et al [59] described the design of a digital comic maker for children with autism spectrum disorders. The design approach led to finding different problem perceptions among stakeholders and added value to the acceptability of the developed innovation by exploring and sharing different sensemaking processes from different stakeholder perspectives. Milton et al [66] reported on the use of participatory design processes to customize and configure a technological solution for a nontraditional mental health service for people with eating disorders and body image issues. The authors reported that participatory design processes enabled a collaborative approach to the customization and configuration of the platform. Knight et al [14] described a participatory design process for developing virtual reality therapy for people with psychosis, which facilitated collaboration between expert groups, including individuals with lived experience of psychosis. This approach led to design and innovation within a shared understanding of limitations and evidence-based design. Fonseka et al [58] adapted a clinical guideline through a co-design approach to an accessible version for patients and families, emphasizing the importance of informed discussion and partnership between people with lived experience and researchers and designers.

#### Design for Identifying the Needs of Service Users and People With Lived Experience

There were 9 studies that reported added value in identifying user needs [43,46-48,56,57,60,72,73]. Schmitt and Yarosh [47] reported on the value of participatory design workshops in aiding the recovery of women with substance use disorders. The workshops provided new insights into the participants’ needs, emphasizing the importance of anonymity and safety in recovery. Owens et al [43] used a participatory approach to develop a SMS text messaging intervention to reduce repetitive self-harm and found that people with lived experience preferred individualized texting instead of a generic one-size-fits-all approach. Grim et al [46] used participatory design processes to develop a web-based system and found that incorporating user perspectives can tailor an innovation to the needs of the user group. Maathuis et al [57] used a value-sensitive design approach to identify potential value conflicts in the design of an internet-based quality-of-life tool for people with mental health problems. The study showed how the approach made it possible to identify and anticipate potential value conflicts and patients’ needs in the design. McClelland and Fitzgerald [48] used a co-design approach to develop a mobile app for service users involved in the early intervention services for psychosis. The authors described key needs that emerged from service user involvement, and they incorporated those needs into the app prototype design, emphasizing that early input is essential to design flexible and relevant contents that meets user needs.

Stawarz et al [60] identified new perspectives and requirements by exploring service users’ needs using user-centered design methods. As a result, the authors formulated 12 design considerations for the integrated delivery of cognitive behavioral therapy for people with depression. Jenness et al [73] used a human-centered design process to develop an app to provide an evidence-based psychosocial intervention for teenagers with depression, and they found that it helped teenagers reduce avoidance. Involving service users in the design process led to design changes based on their needs, such as personalizing the timing and frequency of logging reminders based on participants’ routines and times when they felt most comfortable. Realpe et al [56] found that the co-design process with young people with psychosis led to unexpected insights regarding their needs and wishes, which challenged the researchers’ understanding of what service users needed during recovery. For example, the approach revealed that young people preferred designs that resembled familiar environments with an urban feel, such as local therapeutic spaces or classrooms, rather than stereotypical places of leisure. Illarregi et al [72] focused on the experience of 1 service user in a co-design study, providing insights into how the design activity can support recovery.

#### Design for Empowering Service Users and People With Lived Experience as Resourceful Actors

In total, 6 studies reported the added values of empowering participants as resourceful actors through design approaches [6,44,45,50,62,63]. Gammon et al [44] highlighted the importance of involving service users in the design process of a web-based tool for assisted self-help in mental health. Their study showed that involving service users through design approaches could reveal gaps in relevance between mainstream research and service users’ interests, and the involvement fosters commitment to follow through in the implementation and research phases. The authors found that the approach stimulated to involve service users, as no one is more capable of conveying the intentions and functionalities of the innovation than the service users. Flobak et al [62] reported that the participatory design could balance the perspectives of people with attention-deficit/hyperactivity disorder and clinicians, leading to mutual learning. The authors noted that in their study, participants were not only experts on what they liked or disliked but also very knowledgeable about ADHD, as they had first-hand experience and tacit knowledge of the ADHD diagnosis. This experiential knowledge was initially unavailable to the clinicians and deemed very important for the design process of the intervention. van der Meer et al [63] found that service users with complex mental health problems could meaningfully participate in and contribute to understanding the problem and reflecting on the form and content of a psychosocial intervention. Even service users who were less able to express their needs and desires regarding the content or design of the intervention could still identify the factors they felt were important to consider, and these factors played crucial roles in developing the psychosocial intervention.

Nakarada-Kordic et al [6] reported that service users identified unique needs and interests that differed significantly from those identified by clinicians. Creative participatory methods meaningfully involved young people experiencing psychosis in the process. The study emphasized the importance of treating young people as equal partners and acknowledging them as experts in their own conditions and experiences. The design workshops showed the authors that the unique needs and interests of the participants posed a greater challenge than their severe mental health issues. Terp et al [45] used a design approach to develop a mobile phone tool that enabled young adults with schizophrenia to be actively involved in designing more participatory mental health services. The authors emphasized the importance of giving the community of practice a name and body from the beginning of the design process so that young adults with schizophrenia feel included solely as experts in their own experience and not as patients. The community of practice functioned as an “identity changer” from “receiver” to “giver,” or from a patient in need to a designer of the need. Terp et al [50] concluded in a follow-up study among young adults with schizophrenia that close collaboration between designers, researchers, and users ensured that the app met the target audience’s needs.

### Reported Challenges

The involvement of service users and people with lived experience through design approaches presents several challenges. Participants’ continued participation throughout the study is challenging due to changing mental states, economic constraints, recruitment and panel size, and difficulty in reaching participants [43,53,54,58,64,70,73]. The iterative nature of design makes it difficult to record dynamic individual small group work [43,63,66], and using a design approach is reported to be time-consuming [44,46,48,56]. Providing a safe space for service users and people with lived experience is not only important but also challenging [45,53,68], and designers must be aware of the trade-off between preserving authenticity and reinforcing the stigmatizing characteristics of mental health [62]. Balancing insights from design and medical science can be challenging, as stakeholder recommendations may be inconsistent with evidence-based practices or theory-based principles of change [49,57]. Managing design processes in medical environments is challenging because of the disruptive nature of co-design formats and methods, difficulty in finding a balance between easy navigation in the app and meeting participant requests for additional features, and logistics of capturing inputs from multiple concurrent discussions [6,65,67].

## Discussion

### Principal Findings

This systematic review highlights the advantages of involving service users and people with lived experience through design approaches for innovation in mental health care. Innovation projects in mental health care involve service users and people with lived experience in design processes, with the first publication occurring in 2010. Most studies involved service users, mostly adults. Co-design is the most frequently used design approach. Innovation primarily aimed to develop digital interventions that promote recovery. Participants were often involved as cothinkers and advisers, and sometimes as partners, but never as decision makers. We identified 4 themes in which we categorized the added values of design approaches, namely, design for tailoring and testing interventions and applications, to increase engagement and collaboration in the development of interventions, to identify the needs of service users and people with lived experience, and to empower them as resourceful actors. The challenges reported were maintaining participants’ continued participation throughout the study, managing the iterative nature of design, providing a safe space, balancing insights from design and medical science, and navigating design processes in medical environments.

Many of the included studies emphasized the end results rather than the design processes. Moreover, most studies did not describe whether and how they evaluated the participants’ experiences in the design approach. This can be explained by the fact that the objectives of these studies were not primarily aimed at reporting on the involvement of the participants and their experiences but rather on the development of the interventions or applications. In the studies that we categorized as promoting the partner role, co-design was the design approach that was used most often. This shows the promise of the co-design approach for future innovation projects in mental health care, particularly when significant participation of service users and people with lived experience is required. In line with earlier research, further advantages can be obtained by engaging end users early in the design processes [74]. Many studies reported added values on various topics that centered on the engagement and collaboration in innovation projects. However, based on the findings in this review, this collaboration focused usually on the goals of the researchers and not on the benefits that the design approaches can have for service users and people with lived experience. Although multiple challenges were discovered, these seem to be manageable and can be overcome with more methodological and organizational “design mindedness,” in line with earlier research on the challenges of design in health [75-79].

### Comparison With Previous Findings

Several studies overlap the scope of this systematic review. In an exploratory mapping review, Vial et al [33] examined the literature to understand how human-centered design is considered in e-mental health intervention research. Their findings show that the included studies relied very little on designers and design research, with limited involvement of end users in the design process. In our review, design approaches were used in most studies as a form of traditional research as opposed to how these approaches are described in the design literature, and the level of involvement of service users and people with lived experience was typically low for design processes. The results of our systematic review are also consistent with the those of 4 other reviews [80-83]: co-design processes involving service users and people with lived experience contribute to person-centered innovation; as a result, design can help shift the power balance in favor of susceptible populations. However, the implementation and explanation of design requirements are lacking, and the reporting offers no substantial ground for definitive conclusions. In our review, many studies that created an intervention or application did not describe a design rationale, whereas previous research has shown that this is essential in determining the thoughts and foundations of an intervention to enable researchers to build on it [74,84,85].

This systematic review leverages the existing literature to show the potential of design approaches to empower service users and people with lived experience as resourceful actors in innovation processes in mental health care. This finding suggests that high levels of involvement through design may not only have beneficial outcomes for the greater good of the developed product but also contribute to the benefits of the participants involved. Although this value could be miniscule, it is an effect that has not been emphasized previously, exhibiting some therapeutic potential. The studies wherein the design approaches display high levels of involvement seem to foster equal cooperation and shared objectives, which share similarities with the fundamental elements of therapy, such as therapeutic alliances and expectation-rich therapeutic rituals [86]. In addition, using design approaches seems to align with the need to prioritize the use of participatory methods that facilitate the collaboration with service users, empowering them to meaningfully influence design decisions as important stakeholders [87].

### Strengths and Limitations

The purpose of this systematic review is to provide an overview of the involvement of service users and people with lived experience in mental health care services through design approaches and to identify its advantages. Therefore, we used broad search terms to include as many studies as possible that covered the topic and provide a complete overview of its advantages. To ensure methodological quality, all the included studies were peer reviewed and published in academic journals. To analyze the results, we used a thematic synthesis to have the categories emerge from the data. Although this approach is appropriate for finding new data, the results of the synthesis may appear different from an a priori theoretically driven approach [42]. However, a priori themes could have resulted in overlooking advantages that did not fit within these established categories, such as the potential that design approaches have for empowering service users as resourceful actors. This could have limited the overview of the design approaches and their advantages, which was the main aim of this systematic review.

### Future Research

Although the current emphasis on empowerment, autonomy, involvement, and participation in design-based innovation is a positive development, sharing numerous principles with the recovery movement [88], the included studies provide little information on what they consider involvement and participation, how they intend it, and how they integrate these principles into their methodology. Given that most studies that were categorized as promoting the partner role were also the studies that were categorized as empowering, we recommend that future studies examine what partner and decision maker roles can accomplish to yield therapeutic and recovery outcomes. Furthermore, we suggest that future design studies that focus on innovation in mental health care use a framework that helps to report engagement with service users and people with lived experience in a systemic and clear manner, such as the Involvement Matrix [38]. Our review identified relevant data on the involvement of service users and people with lived experience, but these data were reported in various sections of the papers, were often brief, and lacked detail, corresponding with previous research on patient and public involvement in health and social care research [89]. Involvement frameworks can be valuable when patient-researcher partnerships are led by researchers with little experience involving service users in research [90]. Other directions for future research are examining the different uses of experiential knowledge and its usefulness for design research, investigating whether the use of experiential knowledge by mental health professionals can be beneficial, and exploring how research can benefit from cocreation and how these impact service users, people with lived experience, and mental health professionals.

### Conclusions

This systematic review provides an overview of the involvement of service users and people with lived experience in mental health care services through design approaches and their advantages. The results show that design approaches add value to tailoring and testing applications, interventions, digital applications, and digital interventions; improving engagement and collaboration in the development of interventions; identifying the needs of stakeholders; and empowering service users and people with lived experience as resourceful actors. The challenges that were identified are maintaining participants’ continued participation throughout the study, managing the iterative nature of design, providing a safe space, balancing insights from design and medical science, and navigating design processes in medical environments. Of all the design approaches, co-design was identified as achieving the highest level of involvement and can be useful for innovation in mental health care, wherein significant participation of service users and people with lived experience is required. Future studies using design approaches in mental health care are recommended to systematically and clearly report on involvement and collaboration.

## Appendix

Multimedia Appendix 1 Overview of search terms.

## Abbreviations

PRISMA: Preferred Reporting Items for Systematic Reviews and Meta-Analyses
RQ: research question
