# Supplementary material for: The Involvement of Service Users and People With Lived Experience in Mental Health Care Innovation Through Design: Systematic Review
Source: JMIR Ment Health. 2023 Jul 25;10:e46590. doi: 10.2196/46590 (PMC10410372; doi:10.2196/46590)
Supplement: Multimedia Appendix 1 [file mental_v10i1e46590_app1.docx]

**Reporting search strategy**

**The searches below yielded the following results on 25 October 2022; PubMed 949 references, Embase 407 references, PsychInfo 694 references, Scopus 1473 references and Web of Science 962 references.**For deduplicaton we used the Amsterdam efficient deduplication (AED) method manual. (2020), René Otten, R., Linda Schoonmade, Ralph de Vries from the Universiteitsbibliotheek, department Research Support Vrije Universiteit Amsterdam. <https://zenodo.org/record/3741885#.X2tPeufgpaQ>

**PubMed.gov 23 October 2022
(("Mental Health"[Mesh] OR mental-health[tiab] OR mental-hygiene[tiab] OR mental-care[tiab] OR "Psychiatry"[Mesh] OR psychiatr*[tiab]) AND (user-centered development*[tiab] OR user-centred design*[tiab] OR user-centred development*[tiab] OR user-centric design*[tiab] OR user-driven design*[tiab] OR user-driven development*[tiab] OR "User-Centered Design"[Mesh] OR user-centered-design*[tiab] OR usability-testing[tiab] OR Co-design*[tiab] OR Participatory-design*[tiab] OR Experience-based-co-design*[tiab] OR Interaction-design*[tiab] OR Service-design*[tiab] OR Systemic-design*[tiab] OR Patient-centered-design*[tiab] OR Human-centered-design*[tiab] OR Value-sensitive-design*[tiab] OR Design-thinking[tiab] OR Design-research*[tiab] OR Design-method*[tiab] OR Design-session*[tiab] OR design-approach*[tiab] OR design-principle*[tiab] OR design-choice*[tiab] OR universal-design*[tiab] OR Creativ-method*[tiab] OR Creative-session*[tiab] OR Generative-design*[tiab] OR Generative-method*[tiab] OR Generative-session*[tiab] OR Iterative-design*[tiab] OR Design-driven-innovation*[tiab] OR Speculative-design*[tiab] OR Critical-design*[tiab] OR Discursive-design*[tiab] OR Product-design*[tiab])) NOT ("Research Design"[Mesh] OR research-design[tiab] OR research-designs[tiab] OR research-protocol*[tiab] OR research-instrument*[tiab] OR study-design[tiab] OR study-designs[tiab] OR research-method*[tiab] OR methodology[tiab] OR methodological-research[tiab])
Results: 949 references**

**Search key:
MeSH: medical subject heading
[tiab]: searches in title or abstract*** used For variable truncation (i.e., one or more letters) - e.g. cat* retrieves ‘cat’, ‘cats’, ‘catalyst’, ‘catastrophe’

**Web of Science Core collection (**Science Citation Index Expanded 2010 - present,The Netherlands Social Sciences Citation Index 2010-present, Arts & Humanities Citation Index 2010-present Emerging Sources Citation Index (ESCI) 2010-present) **25 october 2022**

**TS=(** user-centered development* OR user-centred design* OR user-centred development* OR user-centric design* OR user-driven design* OR user-driven development* OR **user-centered-design* OR usability-testing OR Co-design* OR Participatory-design* OR Experience-based-co-design* OR Interaction-design* OR Service-design* OR Systemic-design* OR Patient-centered-design* OR Human-centered-design* OR Value-sensitive-design* OR Design-thinking OR Design-research* OR Design-method* OR Design-session* OR design-approach* OR design-principle* OR design-choice* OR universal-design* OR Creative-method* OR Creative-session* OR Generative-design* OR Generative-method* OR Generative-session* OR Iterative-design* OR Design-driven-innovation* OR Speculative-design* OR Critical-design* OR Discursive-design* OR Product-design*) AND TS=(mental-health OR mental-hygiene OR mental-care OR psychiatr*) NOT TS=(research-design OR research-designs OR research-protocol* OR research-instrument* OR study-design OR study-designs OR research-method* OR methodology OR methodological-research)
Results: 1012 references, excluded 50 meeting abstracts, proceeding papers, editorials, book chapters and letters.
Results: 962 references, 367 references** after removing results from PubMed search.

**Search key: TS= topic, which includes title, abstract, author supplied keywords and Web of Science Keyword Plus**

**Scopus Scopus.com 25 october 2022**

TITLE-ABS-KEY (user-centered-development* OR user-centred-design* OR user-centred-development* OR user-centric-design* OR user-driven-design* OR user-driven-development* OR user-centered-design* OR usability-testing OR co-design* OR participatory-design* OR experience-based-co-design* OR interaction-design* OR service-design* OR systemic-design* OR patient-centered-design* OR human-centered-design* OR value-sensitive-design* OR design-thinking OR design-research* OR design-method* OR design-session* OR design-approach* OR design-principle* OR design-choice* OR universal-design* OR creative-method* OR creative-session* OR generative-design* OR generative-method* OR generative-session* OR iterative-design* OR design-driven-innovation* OR speculative-design* OR critical-design* OR discursive-design* OR product-design* ) AND TITLE-ABS-KEY ( mental-health OR mental-hygiene OR mental-care OR psychiatr* ) AND NOT TITLE-ABS-KEY **(research-design OR research-designs OR research-protocol* OR research-instrument* OR study-design OR study-designs OR research-method* OR methodology OR methodological-research)**
**Results: 1773 references, 896 references** after removing results from PubMed search.

Search key: TITLE-ABS-KEY = searches in title, abstract and author supplied keywords, EMTREE subject headings, other keywords, trade names and chemical names.
* used For variable truncation (i.e., one or more letters) - e.g. cat* retrieves ‘cat’, ‘cats’, ‘catalyst’, ‘catastrophe’

**APA PsycInfo via Ebsco host 25 october 2022**

( ( TI (user-centered-development* OR user-centred-design OR user-centred-designs OR user-centred-development* OR user-centric-design OR user-centric-designs OR user-driven-designs OR user-driven-design OR user-driven-development* OR user-centered-design* OR usability-testing OR co-design* OR participatory-design* OR experience-based-co-design* OR interaction-design* OR service-design* OR systemic-design* OR patient-centered-design* OR human-centered-design* OR value-sensitive-design* OR design-thinking OR design-research* OR design-method* OR design-session* OR design-approach* OR design-principle* OR design-choice* OR universal-design* OR creative-method* OR creative-session* OR generative-design* OR generative-method* OR generative-session* OR iterative-design* OR design-driven-innovation* OR speculative-design* OR critical-design* OR discursive-design* OR product-design* ) ) OR AB ( ( user-centered-development* OR user-centred-design OR user-centred-designs OR user-centred-development* OR user-centric-design OR user-centric-designs OR user-driven-designs OR user-driven-design OR user-driven-development* OR user-centered-design* OR usability-testing OR co-design* OR participatory-design* OR experience-based-co-design* OR interaction-design* OR service-design* OR systemic-design* OR patient-centered-design* OR human-centered-design* OR value-sensitive-design* OR design-thinking OR design-research* OR design-method* OR design-session* OR design-approach* OR design-principle* OR design-choice* OR universal-design* OR creative-method* OR creative-session* OR generative-design* OR generative-method* OR generative-session* OR iterative-design* OR design-driven-innovation* OR speculative-design* OR critical-design* OR discursive-design* OR product-design* ) ) OR KW ( (user-centered-development* OR user-centred-design OR user-centred-designs OR user-centred-development* OR user-centric-design OR user-centric-designs OR user-driven-designs OR user-driven-design OR user-driven-development* OR user-centered-design* OR usability-testing OR co-design* OR participatory-design* OR experience-based-co-design* OR interaction-design* OR service-design* OR systemic-design* OR patient-centered-design* OR human-centered-design* OR value-sensitive-design* OR design-thinking OR design-research* OR design-method* OR design-session* OR design-approach* OR design-principle* OR design-choice* OR universal-design* OR creative-method* OR creative-session* OR generative-design* OR generative-method* OR generative-session* OR iterative-design* OR design-driven-innovation* OR speculative-design* OR critical-design* OR discursive-design* OR product-design* ) ) ) AND ( TI ( mental-health OR mental-hygiene OR mental-care OR psychiatr* ) OR AB ( mental-health OR mental-hygiene OR mental-care OR psychiatr* ) OR KW ( mental-health OR mental-hygiene OR mental-care OR psychiatr* ) ) NOT **(research-design OR research-designs OR research-protocol* OR research-instrument* OR study-design OR study-designs OR research-method* OR methodology OR methodological-research)**
Excluded: dissertations and books,
**Results: 694 references, 414 references** after removing results from PubMed search.

Search key:
TI= title, AB= abstract and KW= Searches for keywords in the uncontrolled content description of the document
* used For variable truncation (i.e., one or more letters) - e.g. cat* retrieves ‘cat’, ‘cats’, ‘catalyst’, ‘catastrophe’

**Embase.com**('mental health'/exp OR 'mental care':ab,ti,kw OR 'mental health':ab,ti,kw OR 'mental hygiene':ab,ti,kw OR 'mental condition':ab,ti,kw OR 'psychic health':ab,ti,kw OR 'mental state':ab,ti,kw OR 'mental status':ab,ti,kw OR 'psychiatry'/exp OR psychiatr*:ab,ti,kw) AND ('user-centered design'/exp OR 'user centered development*':ab,ti,kw OR 'user centred design*':ab,ti,kw OR 'user centred development*':ab,ti,kw OR 'user centric design*':ab,ti,kw OR 'user driven design*':ab,ti,kw OR 'user driven development*':ab,ti,kw OR 'user centered design*':ab,ti,kw OR 'usability testing':ab,ti,kw OR 'co design*':ab,ti,kw OR 'participatory design*':ab,ti,kw OR 'experience based co design*':ab,ti,kw OR 'interaction design*':ab,ti,kw OR 'service design*':ab,ti,kw OR 'systemic design*':ab,ti,kw OR 'patient centered design*':ab,ti,kw OR 'human centered design*':ab,ti,kw OR 'value sensitive design*':ab,ti,kw OR 'design thinking':ab,ti,kw OR 'design research*':ab,ti,kw OR 'design method*':ab,ti,kw OR 'design session*':ab,ti,kw OR 'design approach*':ab,ti,kw OR 'design principle*':ab,ti,kw OR 'design choice*':ab,ti,kw OR 'universal design*':ab,ti,kw OR 'creativ method*':ab,ti,kw OR 'creative session*':ab,ti,kw OR 'generative design*':ab,ti,kw OR 'generative method*':ab,ti,kw OR 'generative session*':ab,ti,kw OR 'iterative design*':ab,ti,kw OR 'design driven innovation*':ab,ti,kw OR 'speculative design*':ab,ti,kw OR 'critical design*':ab,ti,kw OR 'discursive design*':ab,ti,kw OR 'product design*':ab,ti,kw) NOT ('methodology'/exp OR 'methodology' OR 'methodological research':ab,ti,kw OR 'research method*':ab,ti,kw OR 'research protocol*':ab,ti,kw OR 'research instrument':ab,ti,kw OR 'research design':ab,ti,kw OR 'research designs':ab,ti,kw OR 'study design':ab,ti,kw OR 'study designs':ab,ti,kw) AND ('article'/it OR 'article in press'/it OR 'erratum'/it OR 'review'/it OR 'short survey'/it)

**Results: 407 references, 132 references** after removing results from PubMed search.

Search key:
Ab, ti, kw searches in abstract, title and author supplied keywords
/exp searches Emtree preferred indexing term and then searches for the related narrower or child terms.
* used For variable truncation (i.e., one or more letters) - e.g. cat* retrieves ‘cat’, ‘cats’, ‘catalyst’, ‘catastrophe’

**Database Flow chart**Total results imported in Zotero https://www.zotero.org/ for deduplicaton 2758.
 **PubMed 949 references
Embase 132 references
PsychInfo 414 references
Scopus 896 references
Web of Science 367 references

Total references exported to Rayyan https://www.rayyan.ai/ after deduplicaton for screening on title, abstract and full text: 2013.

Journal searching 25 october 2022
We identified two journals that publish on the intersection of health and design, Design for Health and The Journal of Health Design. Because both journals publish design related articles, we used the search key:

 ‘mental health OR psychiatry’

We identified 65 articles in Design for Health and 6 articles in The Journal of Health Design.
Results: 65 references, 54 references after deduplication.**
